# Prescription of guideline‐directed medical therapy in heart failure: impact on mortality and readmission

**DOI:** 10.1002/ehf2.15280

**Published:** 2025-04-29

**Authors:** Martin Möckel, Samipa Pudasaini, Kristina Feldmann, Henning Thomas Baberg, Benny Levenson, Jürgen Malzahn, Thomas Mansky, Guido Michels, Christian Günster, Elke Jeschke

**Affiliations:** ^1^ Department of Emergency and Acute Medicine, Campus Virchow‐Klinikum and Campus Charité Mitte Charité – Universitätsmedizin Berlin, Corporate Member of Freie Universität and Humboldt‐Universität zu Berlin Berlin Germany; ^2^ Department of Cardiology and Nephrology Helios Klinikum, Berlin‐Buch and Medical School Berlin Berlin Germany; ^3^ German Society of Cardiologists in Private Practise (BNK) Berlin Germany; ^4^ Federal Association of the Local Health Care Funds (AOK) Berlin Germany; ^5^ Faculty of Economics and Management Division of Structural Development and Quality Management in Healthcare, Technische Universität Berlin Berlin Germany; ^6^ Department of Emergency Medicine Hospital of the Barmherzige Brüder Trier Germany; ^7^ Research Institute of the Local Health Care Funds (WIdO) Berlin Germany

**Keywords:** Heart failure, Drug therapy, Guideline adherence, Mortality, Readmission

## Abstract

**Aims:**

The 2021 European heart failure (HF) guidelines recommend the combination of four drugs as a standard therapy (angiotensin‐converting enzyme inhibitor [ACEI]/angiotensin receptor blocker [ARB]/angiotensin receptor‐neprilysin inhibitor [ARNI]; beta‐blocker (BB); mineralocorticoid receptor antagonist [MRA]; sodium‐glucose co‐transporter 2 inhibitor [SGLT2i]) in patients with heart failure and reduced ejection fraction (HFrEF). We investigated if the use of this combined treatment (as opposed to the outdated two‐drug ACEI/ARB and BB therapy) yields a favourable outcome regarding mortality and readmission and evaluated whether an increase in adoption of the newly endorsed therapy can already be observed in clinical routine.

**Methods and results:**

We included anonymous data from all patients who were insured at Germany’s largest health insurer (Allgemeine Ortskrankenkasse [AOK]) and had a claims record for hospitalization (2019–2021) with the main diagnosis of HF. Mortality and readmission within 91–365 days following the index stay were analysed, and the impact of medication on outcome was compared. 315 342 cases of hospitalization due to HF were included (median 80 years [IQR 72–86], 53.7% female). HF drug prescription rates were as follows: ACEI 46.3%, ARB 31.8%, ARNI 12.1%, BB 80.9%, MRA 35.6%, SGLT2i 7.3%. Treatment combinations were prescribed in 35.9% (two‐drug) and 3.7% (four‐drug). Total mortality was 18.0%, all‐cause readmission 32.0%, and HF readmission 16.0%. Mortality risk was significantly lower (adjusted HR = 0.92 [95% CI 0.86–0.97]) with the four‐ versus two‐drug treatment. Kaplan–Meier survival was 88.2% for the four‐drug therapy [95% CI: 87.6%–88.8%] and 83.1% for the two‐drug therapy [95% CI: 82.9%–83.3%]). Similar benefits were visible for the readmission rates due to all causes (HR = 0.76 [0.73–0.80]) and readmission due to HF (HR = 0.90 [0.85–0.95]).

**Conclusions:**

Our study suggests that the newly recommended four‐drug therapy may lead to lower mortality and readmission rates compared to the outdated two‐drug therapy. However, the overall adoption of the four‐drug therapy remains limited.

## Graphical abstract

Routine data analysis showing a better outcome regarding mortality and readmission in HF patients when being prescribed the current four‐drug treatment (vs. the outdated two‐drug therapy) 91–365 days after index hospital stay.

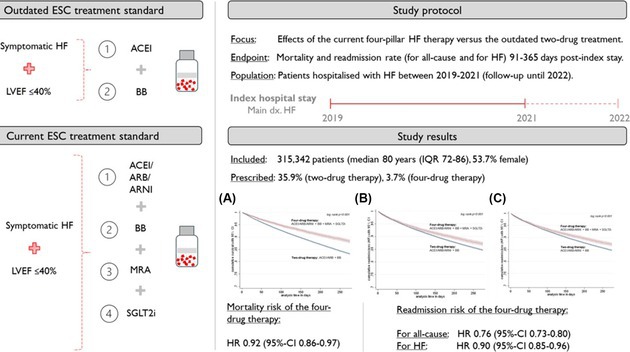

## Introduction

Chronic heart failure (HF) is one of the most challenging diseases for affected patients, their caregivers and healthcare systems. According to the global burden of disease study, the worldwide prevalence is estimated at 56.2 million cases with relevant ethnical disparities and assumed regional underreporting.^1^ Adequate therapeutic strategies and guideline adherence are key to improving patients' health‐related quality of life, decelerating disease progression and reducing mortality.^2^ Routine data may serve as quality indicator for adherence to guidelines in the patient population.^3^

Until 2012, the basic pharmacological therapy for patients with symptomatic HF and a reduced left ventricular ejection fraction (LVEF) of ≤40% implied the combination of an angiotensin‐converting enzyme inhibitor (ACEI) or an angiotensin receptor blocker (ARB) and a beta‐blocker (BB).^4^ Both were prescribed by titrating them to the maximum tolerated evidence‐based dose. Further significant HF drug classes already existed but were only used in addition or as an alternative to the primary medication.^4^ These include mineralocorticoid receptor antagonists (MRAs), which have the potential to decelerate cardiac remodelling and reduce hypervolemia.^5^ MRAs were, thus, included to the Ia 2016 guideline recommendations by the European Society of Cardiology (ESC) for patients with a LVEF ≤35% and under consideration of their renal function and the serum potassium levels.^6^ Before, addition of MRA was recommended only in patients with persistent symptoms under standard therapy.^7^ Over time, promising outcomes were likewise reported for angiotensin receptor‐neprilysin inhibitors (ARNIs),^8^ as well as for sodium‐glucose co‐transporter 2 inhibitors (SGLT2is), a medication class that showed significant benefits when being used in (diabetic/non‐diabetic) patients who kept presenting with a LVEF ≤40% and HF symptoms despite receiving optimal medical therapy.^9^ Based on these findings, the new 2021 guidelines implemented fundamental changes in the recommendation of primary HF drug therapy. A simultaneous start of now four main HF medication groups and a rapid titration of all are suggested to be used as the gold standard practice.^2^ The quadruple therapy includes firstly an ACEI, an ARB, or an ARNI; secondly, a BB; thirdly, a MRA; and, fourthly, a SGLT2i.^2^ All drug types have been proven to reduce rehospitalization and mortality rates as well as the symptomatic burden itself.^2^, ^5^, ^9^, ^10^, ^11^ In addition, the use of loop diuretics was suggested in the lowest possible dose if needed to treat fluid retention and maintain euvolemia.^12^

The constant progress and adaptation in drug therapy is primarily based on findings of randomized controlled trials (RCTs), which analysed outcomes of single HF drug prescriptions versus placebo/alternative medication in affected patients.^5^, ^9^, ^10^, ^11^ Studies researching the effects of combined pharmacological treatments also exist; however, they mainly focus on therapies that do not correspond to the current standard.^13^ The outcome of the newly recommended guideline‐directed medical therapy (GDMT) has, thus, rarely been investigated. An exception is the work of Vaduganathan *et al*., who compared treatment effects of the quadruple medication (specifically ARNIs, BB, MRA, and SGLT2i) with the old double therapy standard in a cross‐trial analysis of three existing RCTs (EMPHASIS‐HF, PARADIGM‐HF, and DAPA‐HF). Their study indicated a significant superiority of the four‐drug combination regarding cardiovascular death and readmission.^14^ Such specific comparative investigations are, however, not yet performed in a real‐world setting to evaluate if RCT and cross‐trial outcome results can be reproduced. This was the primary objective of our work. To do so, we used a large set of routinely collected data, a method that was successfully applied in previous literature.^15^ Secondarily, GDMT utilization was assessed to evaluate current prescription patterns and the extent of guideline implementation.

## Methods

### Study design and study population

For this observational routine data study, we used anonymized nationwide administrative claims data of the Allgemeine Ortskrankenkasse (AOK) of hospitalized HF patients (with reduced ejection fraction). The AOK provides healthcare insurance for approximately 30% of the German population and is the largest nationwide provider of statutory healthcare insurance in Germany.^16^ Everyone is allowed to enrol in the AOK regardless of factors such as age, comorbidity, income, or type of employment; therefore, AOK patients presumably represent the broad majority of German inhabitants across all social strata and milieus.

We evaluated billing data for inpatient treatment, including diagnoses, as well as patient data regarding age, gender, insurance status (i.e. continued/terminated AOK membership) and mortality. Diagnoses were encoded according to the International Classification of Diseases (ICD‐10). Procedures were documented using the German version of the International Classification of Procedures in Medicine (ICPM), the OPS code. Healthcare and health insurance providers jointly issue binding guidelines for the coding of diagnoses and procedures in German hospitals. Hospital billing data are thoroughly checked by the Medical Review Board of the Health Insurance Funds and are returned to hospitals for correction, if necessary.

We included anonymized data from all patients with a claims record for hospitalization and a main diagnosis of HF (I11.0, I13.0, I13.2, I50) from 2019 to 2021. Exclusion diagnoses were congenital anomalies (Q20–Q28), transplantation (T86, Z09.80, Z94.1, Z94.3), and asthma (J45, J46). Also excluded were patients aged <30 years, as well as those with a record of hospitalization due to HF in the previous year, and incomplete medication data for the first 90 days after index hospital stay. Additionally, HF patients who tested positive for coronavirus disease 19 (COVID‐19) at index hospitalization were likewise excluded. Exclusions were done to reduce heterogeneity of the data set and clearly distinguish index from follow‐up events (previous year exclusion).

### Exposure

Primarily, the use of ACEIs, ARBs, BBs, ARNIs, MRAs, and SGLT2is was analysed in the time period between 91 and 365 days after index hospitalization. The time period between 1 and 90 days after hospitalization was excluded because hospitalization often leads to the prescription of new medication and we aimed to make sure that the new medication is able to exert its effects during a significant time period.

### Outcomes

The primary outcome was all‐cause mortality within 91––365 days after discharge (follow‐up until 2022). Secondary endpoints were rehospitalization for any cause and rehospitalization due to the diagnosis HF in the same time span. The outcomes were analysed depending on HF drug prescription. Specifically, patients who received the outdated two‐drug treatment (ACEI/ARB and BB) were compared to those who were being prescribed the new four‐drug standard therapy (ACEI/ARB/ARNI, BB, MRA, SGLT2i). Numbers of three‐drug prescriptions are also reported; however, they are not in the foreground regarding the main group comparison. This would have needed additional data on the patients' LVEF, renal function, and potassium levels in order to extract the HF patient sub‐group, which actually had a three‐drug indication (+MRA) according to 2016 guidelines, instead of solely the two‐drug standard therapy recommendation from 2012.^6^, ^7^ Overall, a stronger cumulative benefit of all four medication classes was assumed for both outcomes of mortality and readmission. The current data became available in 2023 and, thus, present the most up to date routine data from statutory health insurance.

### Ethical approval

This study was performed by using anonymized health insurance data, a study design that does not require an ethical approval or written informed consent. Recommendations for good clinical practice concerning the use of secondary data sets, as published by the German Working Group on the Collection and Use of Secondary Data, were applied.^17^ The investigation conforms with the principles outlined in the Declaration of Helsinki.

### Statistical analysis

Descriptive statistics including medians, interquartile ranges (IQRs), and proportions were used to describe the study sample. Baseline patient characteristics were compared using Pearson's chi‐squared test for categorical variables.

Unadjusted survival was estimated using the Kaplan–Meier method, with stratification according to different combinations of standard HF drugs. Multivariable Cox proportional hazards models were estimated to evaluate the association of HF medication on the different endpoints while adjusting for patient risk factors. We used cluster robust standard errors in order to account for clustering of patients in hospitals. Models were adjusted for patient age, sex, New York Heart Association (NYHA) stage, coronary heart disease, aortic valve disorder, mitral valve disorder, dilated cardiomyopathy, prior ischaemic stroke or intracranial bleeding (ICB), ventricular tachycardia, chronic obstructive pulmonary disease (COPD), pneumonia, dementia, and Elixhauser comorbidities,^18^ as well as year of admission. Automated backward selection was used. Comorbidities were entered as separate dichotomous variables and patient age as a continuous variable. Adjusted hazard ratios (HR) and 95% confidence intervals (95% CIs) were calculated. The proportional hazards assumption was evaluated by plotting scaled Schoenfeld residuals against time. We also performed a sensitivity analysis by comparing patients with four‐drug therapy to a propensity score‐matched control group with two‐drug therapy. A logistic regression model including all the covariates from the multivariable Cox regression model was used to estimate the propensity score (PS). Nearest neighbour matching (1:1) was performed with the logit‐transformed PS. A calliper of 0.2 was used.^19^ Balance of covariates before and after matching was checked using standardized differences. The treatment effect was computed by means of a Cox proportional hazards model with robust variance estimator in the matched sample.^20^ All analyses were performed using STATA 16.0 (StataCorp LP, College Station, Texas), and a *P* value of <0.05 was considered statistically significant. Patients were censored if their AOK insurance coverage ended before follow‐up was finished. Regarding the readmission analysis, this was also applicable for patients who died during the follow‐up observation time. Therefore, total patient numbers varied by outcome and year of follow‐up.

## Results

### Study cohort

**Figure 1:**
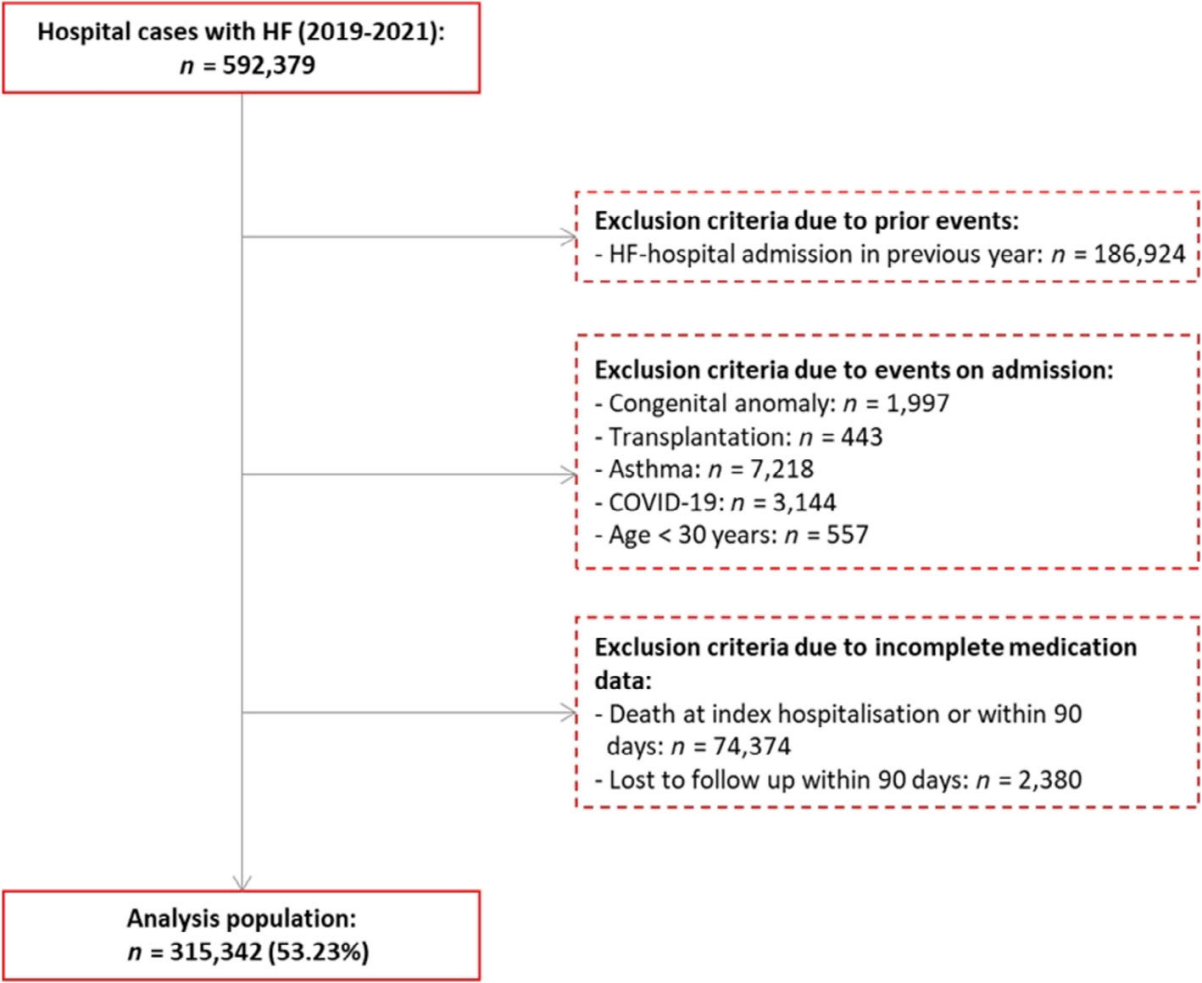
Patient flow chart. Patient flow chart showing the applied inclusion and exclusion criteria that lead to the final study analysis population of *n* = 315 342 out of 592 379 hospital cases that presented with HF as a main diagnosis during index hospital stay in the time span between 2019 and 2021. COVID‐19. coronavirus disease 19; HF. heart failure.

Out of *n* = 592 379 screened HF patients, *n* = 315 342 patients with former stable HF were analysed after applying the exclusion criteria (*Figure* *1*). Basic characteristics of all included patients, as well as stratified by HF drug combination, are visualized in *Table* *1*. Divided by year, 116 907 HF index hospital stays were detected in 2019, 98 650 in 2020, and 99 785 in 2021. All included HF patients had a median age of 80 years (IQR 72–86). Specifically, the median age of patients on a two‐drug therapy was 82 (IQR 75–87) years but it was 71 (IQR 62–80) years for those receiving all four HF medications. In total, 53.7% were female. Divided by the drug combination sub‐group, 60% of patients who received the outdated two‐drug therapy were females, while less than a third (30.8%) of those to whom the newly standardized four‐drug treatment was prescribed were women (see *Table* 1).

**Table 1:** Baseline characteristics

Parameter	Total		Two‐ or four‐drug therapy		Two‐drug therapy		Four‐drug therapy	
	*N*	%	*N*	%	*N*	%	*N*	%
Patients	**315 342**	**100.00%**	**124 894**	**100.00%**	**113 34**	**100.00%**	**11 553**	**100.00%**
Age group									
<66 years	45 429	**14.41%**	15 108	**12.10%**	11 126	**9.82%**	3982	**34.47%**
66–70 years	25 928	**8.22%**	9677	**7.75%**	7974	**7.04%**	1703	**14.74%**
71–75 years	31 180	**9.89%**	12 332	**9.87%**	10 744	**9.48%**	1588	**13.75%**
76–80 years	55 273	**17.53%**	22 358	**17.90%**	20 647	**18.22%**	1711	**14.81%**
81–85 years	77 559	**24.60%**	32 506	**26.03%**	30 751	**27.13%**	1755	**15.19%**
86–90 years	54 363	**17.24%**	22 589	**18.09%**	21 926	**19.35%**	663	**5.74%**
>90 years	25 610	**8.12%**	10 324	**8.27%**	10 173	**8.98%**	151	**1.31%**
Female patients	169 209	**53.66%**	72 358	**57.94%**	68 796	**60.70%**	3562	**30.83%**
Heart failure (I 50)	305 927	**97.01%**	120 173	**96.22%**	108 727	**95.93%**	11 446	**99.07%**
Hypertensive heart and renal disease (I 11)	8645	**2.75%**	4343	**3.48%**	4246	**3.75%**	97	**0.84%**
Hypertensive heart and renal disease with (congestive) heart failure (I 13.0)	138	**0.04%**	65	**0.05%**	64	**0.06%**	1	**0.01%**
Hypertensive heart and renal disease with both (congestive) heart failure and renal failure (I 13.2)	619	**0.20%**	313	**0.25%**	304	**0.27%**	9	**0.08%**
Comorbidity									
Left heart failure								
NYHA I	5356	**1.70%**	2370	**1.90%**	2267	**2.00%**	103	**0.89%**
NYHA II	28 573	**9.06%**	12 275	**9.83%**	11 515	**10.16%**	760	**6.58%**
NYHA III	132 138	**41.90%**	52 522	**42.05%**	47 171	**41.62%**	5351	**46.32%**
NYHA IV	119 082	**37.76%**	45 967	**36.80%**	40 724	**35.93%**	5243	**45.38%**
No specification of NYHA class	30 193	**9.57%**	11 76	**9.42%**	11 664	**10.29%**	96	**0.83%**
Prior known heart failure^a^	136.478	**43.28%**	52 069	**41.69%**	47 035	**41.50%**	5034	**43.57%**
Hypertension	255 978	**81.17%**	106 469	**85.25%**	97 510	**86.03%**	8959	**77.55%**
Atrial fibrillation/flutter	169 401	**53.72%**	69 068	**55.30%**	63 579	**56.10%**	5489	**47.51%**
Coronary heart disease	135 030	**42.82%**	53 605	**42.92%**	46 189	**40.75%**	7416	**64.19%**
Acute myocardial infarction	8408	**2.67%**	3502	**2.80%**	2937	**2.59%**	565	**4.89%**
Prior myocardial infarction	25 018	**7.93%**	9894	**7.92%**	8379	**7.39%**	1515	**13.11%**
Mitral valve disorder	49 962	**15.84%**	18 983	**15.20%**	16 322	**14.40%**	2661	**23.03%**
Aortic valve disorder	34 156	**10.83%**	13.836	**11.08%**	12 799	**11.29%**	1037	**8.98%**
Dilated cardiomyopathy	26 126	**8.28%**	7174	**5.74%**	4404	**3.89%**	2770	**23.98%**
Prior stroke or intracranial bleeding	8678	**2.75%**	3364	**2.69%**	3174	**2.80%**	190	**1.64%**
Ventricular tachycardia	3928	**1.25%**	1251	**1.00%**	816	**0.72%**	435	**3.77%**
Renal failure	147 531	**46.78%**	60 983	**48.83%**	56 869	**50.18%**	4114	**35.61%**
Renal failure requiring dialysis	7115	**2.26%**	3529	**2.83%**	3508	**3.10%**	21	**0.18%**
Diabetes	123 816	**39.26%**	52 596	**42.11%**	44 939	**39.65%**	7657	**66.28%**
COPD	54 146	**17.17%**	19 694	**15.77%**	17 773	**15.68%**	1921	**16.63%**
Pneumonia	37 561	**11.91%**	14 836	**11.88%**	13 567	**11.97%**	1269	**10.98%**
Depression	18 259	**5.79%**	6985	**5.59%**	6512	**5.75%**	473	**4.09%**
Dementia	24 931	**7.91%**	9369	**7.50%**	9095	**8.02%**	274	**2.37%**
Solid tumour without metastasis	6283	**1.99%**	2368	**1.82%**	2110	**1.86%**	158	**1.37%**

Baseline characteristics of all patients included in total as well as divided by the specific combination of therapy (two‐drug vs. four‐drug standard).

COPD, chronic obstructive pulmonary disease; ICD‐10, International Classification of Diseases; NYHA, New York Heart Association Functional Classification.

^a^ Outpatient heart failure diagnosis in the year prior to the index hospitalization.

The majority of all cases had HF that was classified as NYHA stage III (41.9%) or NYHA stage IV (37.8%), and 43.28% of the patients had an outpatient HF diagnosis in the year prior to the index hospitalization. Hypertension was the most common comorbidity by far, with 81.2%, followed by atrial fibrillation/flutter being reported in 53.7% of all patients, renal failure in 46.8% and coronary heart disease in 42.9%. Considerable differences in the presence of comorbidities between the two‐drug and four‐drug sub‐group were observed for coronary heart disease (40.8% vs. 64.1%), dilated cardiomyopathy (3.9% vs. 24.0%), renal failure (50.2% vs. 35.6%), and diabetes (39.7% vs. 66.3%).

### Drug prescription

For the whole observation period, we found prescription of ARNIs in 12.1% and of SGLT2i in 7.3% of the included patients. Further, renin–angiotensin–aldosterone system inhibitors (RAASis) were prescribed in 84.7% of the cases, BB in 80.9%, and both in 70.2%. Separated by RAASi type, 46.3% received an ACEI, 31.8% an ARB, and 35.6% an MRA. Stratified by year (2019/2020/2021), the old standard therapy of solely ACEI/ARB and BB was seen in 37.8%, 36.4%, and 33.2%, representing a decline of this treatment regimen, while prescription of the newly recommended drugs was, by contrast, increasingly observed with 1.3%, 2.4%, and 7.7%, respectively. The most common three‐drug combination consisted of ACEI/ARB/ARNI, BB, and MRA with 26.9%, while ACEI/ARB/ARNI, BB, and SGLT2i were prescribed together in a mere 5.9% of all patient cases (see *Table* *S1*). The remaining HF patients had a prescription of only a single‐drug therapy, alternatively combined therapies or none at all. In addition, the percentage of patients who had a drug prescription of diuretics was reported at 91.7%. The various combination therapies are visualized in *Figure* *S1*.

### Mortality

**Figure 2:**
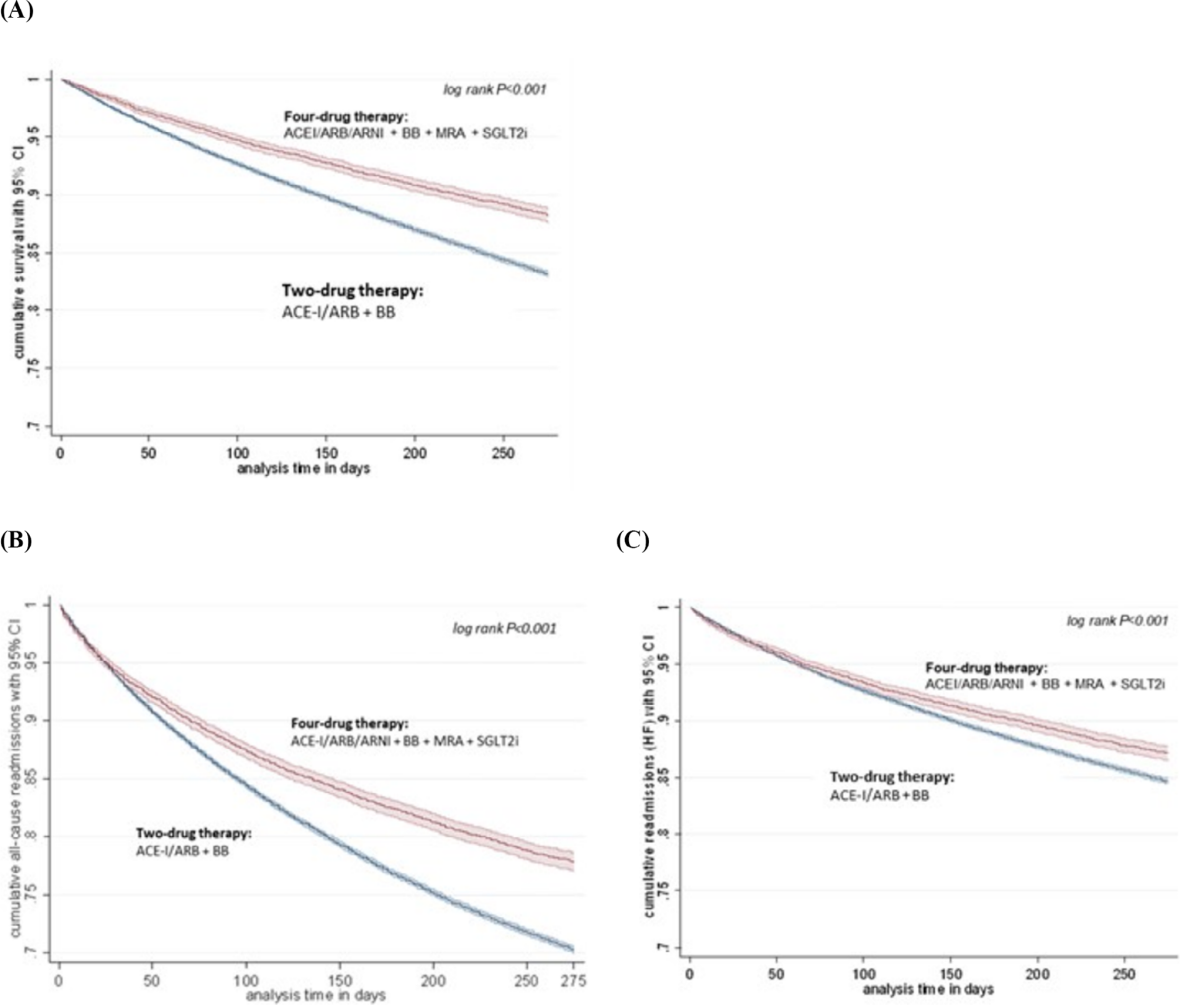
Kaplan–Meier analysis for survival (A) and survival free from readmissions due to (B) all‐cause and (C) HF according to the combination of two or four HF drugs. Kaplan–Meier estimates representing the cumulative survival free from readmissions (for all‐cause and HF) with 95% confidence interval (CI) within 91 and 365 days after index hospitalization. A differentiation according to the use of the newly suggested HF therapy including four drugs (ACEI/ARB/ARNI, BB, MRA, SGLT2i; red curve) versus the use of two HF drugs (ACEI/ARB, BB; blue curve); log‐rank test; significance defined as *P* < 0.05. ACEI, angiotensin‐converting enzyme inhibitor; ARB, angiotensin receptor blocker; ARNI, angiotensin receptor‐neprilysin inhibitor; BB, beta‐blocker; CI, confidence interval; MRA, mineralocorticoid receptor antagonist; SGLT2i, sodium‐glucose co‐transporter 2 inhibitor.

In total, 55 015 of 306 035 (18.0%) included HF patients died 91–365 days after hospital discharge. 9 307 were censored for this analysis. In cases where all four drugs were taken, the survival rate was significantly higher with 88.2% (95% CI 87.6–88.8%), compared to patients who received the outdated two‐drug treatment and presented with a survival rate of 83.1% (95% CI 82.9–83.3%). The corresponding Kaplan–Meier survival curves are presented in *Figure* *2* *A*. The significant difference of *P* < 0.001 remains stable when comparing therapy‐dependent survival rates between females versus males. When analysing survival rates in patients being treated with a two‐drug versus four‐drug therapy depending on their age category, survival only differed in patients aged <66 years (82.0% (95% CI 81.8–82.5%) versus 85.3% (95% CI 84.5–86.1%)), whereas older patients did not show a significant age‐dependent difference in outcome. Also, in patients who had only a combined treatment prescription of three drugs, with the third one being either an MRA or SGLT2i, survival rates were still significantly higher than compared to the outdated two‐drug treatment group (+MRA: 84.4% [95% CI 84.2–84.7%]; +SGLT2i: 88.1% [95% CI 87.6–88.5%]; *P* < 0.01 in both cases).

Multivariable Cox proportional hazards indicate a significantly lower mortality risk when patients had a combined treatment of all four drugs compared to two‐drug therapy (HR = 0.92, 95% CI 0.86–0.97). By contrast, factors like rising age accounted for an increased risk of death in our study population; with an adjusted HR of 2.50 (95% CI 2.32–2.68) in the most strongly represented patient group of 81‐ to 85‐year‐olds. Comorbidities of all kinds, alcohol abuse, and weight loss likewise increased the adjusted HR significantly, while obesity types I and II showed protective effects on the hazard of death. Female sex (HR 0.76, 95% CI 0.74–0.78) was also associated with a lower mortality risk. Details on this are listed in *Table* *2*. The result of the sensitivity analysis in the PS‐matched subsample also shows a beneficial outcome for the four‐drug therapy compared to the two‐drug therapy (HR = 0.86, 95% CI 0.80–0.93). The balance of covariates for patients before and after propensity‐score matching is shown in *Table* *S2*.

**Table 2:** Effect of the four‐drug therapy and other variables on the hazard of mortality 91–365 days after index stay

Risk factor	HR	95% CI
Four‐drug therapy (Ref. two‐drug therapy)	0.92	0.86–0.97
Patient age group (Ref. <66 years)		
66–70 years	1.40	1.29–1.54
71–75 years	1.62	1.50–1.76
76–80 years	1.89	1.75–2.03
81–85 years	2.50	2.32–2.68
86–90 years	3.60	3.35–3.88
>90 years	5.48	5.07–5.92
Female patient	0.76	0.74–0.78
Comorbid conditions		
NYHA stage (Ref. NYHA IV)		
NYHA I	0.55	0.50–0.64
NYHA II	0.59	0.55–0.63
NYHA III	0.86	0.84–0.89
BMI (Ref. BMI < 30 kg/m^2^)		
BMI 30–34	0.75	0.70–0.81
BMI 35–39	0.81	0.74–0.89
BMI 40+	1.05	0.97–1.13
Paralysis	1.21	1.08–1.34
Alcohol abuse	1.26	1.09–1.45
Prior stroke	1.17	1.06–1.29
Psychosis	1.28	1.07–1.54
Valvular disease	1.10	1.06–1.14
Pulmonary circulation disorder	1.11	1.07–1.16
Lymphoma	1.70	1.39–2.08
Metastatic cancer	1.88	1.61–2.19
Solid tumour without metastasis	2.05	1.87–2.24
Rheuma	1.11	1.01–1.22
Coagulopathy	1.11	1.03–1.20
Weight loss	1.31	1.20–1.42
Fluid and electrolyte disorders	1.24	1.20–1.28
Peripheral vascular disorders	1.23	1.18–1.28
Iron deficiency anaemia	1.15	1.09–1.20
Neurological disorders	1.14	1.07–1.22
COPD	1.29	1.24–1.34
Diabetes, uncomplicated	1.13	1.10–1.17
Diabetes, complicated^a^	1.27	1.22–1.32
Renal failure	1.33	1.29–1.37
Dementia	1.64	1.57–1.71
Year of hospital stay (Ref. 2019)		
2020	1.06	1.03–1.10
2021	1.12	1.08–1.16

Model was adjusted for year of hospital stay, patient age group, sex, NYHA stage (IV vs. I, II, III), BMI (<30 kg/m^2^ vs. 30–34, 35–39, and 40+ kg/m^2^), dementia, cardiogenic and unspecified shock, ischaemic stroke, intracerebral bleeding, and concomitant diseases at the index hospitalization by Elixhauser *et al*.^18^ Only significant risk factors are shown.

Abbreviations: CI, confidence interval; COPD, chronic obstructive pulmonary disease; HR, hazard ratio; NYHA, New York Heart Association Functional Classification; Ref., reference.

^a^ Coma, ketoacidosis, vascular disease.

### Rehospitalization

Rehospitalization in the time period between 91 and 365 days post‐discharge was reported in 89 044 out of 277 885 (32.0%) HF cases. 44 439 (16.0%) readmissions were due to a specific diagnosis of HF. 37 457 patients were censored for this analysis due to death or an ending AOK membership prior to the 1‐year follow‐up mark. Kaplan–Meier estimates for survival free from all‐cause readmissions in the observation period were at 70.9% (95% CI 70.7–71.2%). Depending on the type of combination therapy, it varied between 70.2% (95% CI 70.0–70.5%) for the two‐drug combination and 77.8% (95% CI 77.0–78.5%) for the four‐drug therapy, respectively (*P* < 0.001; see *Figure* *2* *B*). This therapy outcome difference was stable for all sex and age categories. Further, the Kaplan–Meier estimate for survival free from rehospitalization due to the specific diagnosis of HF was at 84.9% (95% CI 84.7–85.1%) overall. Stratified by HF drug combination treatment, estimates were measured at 84.7% (95% CI 84.4–84.9%) for the two‐drug sub‐group, and at 87.1% (95% CI 86.5–87.7%) when all four medications were prescribed, showing a significant difference of *P* < 0.001 (see *Figure* *2* *C*). This difference remained visible also when comparing sub‐group outcomes of females versus males (males: *P* = 0.002, females: *P* < 0.001) and age group (<66 years vs. 66–85 years vs. >85 years: all *P* < 0.001).

Multivariable analyses were performed regarding the rehospitalization for all‐cause as well as for HF, specifically. Prescription of the newly endorsed four‐drug HF standard therapy showed a significantly better outcome regarding all‐cause readmission (HR 0.76, 95% CI 0.73–0.80). Likewise, a benefit of the four‐drug therapy on the rehospitalization rate for HF was detectable (HR 0.90, 95% CI 0.85–0.95). When looking at other influencing factors that have a significant impact on all‐cause and HF readmissions, higher age (>66 years), type III obesity, male sex (for HF only), as well as cardiovascular, pulmonary and renal comorbidities increased the readmission risk significantly. By contrast, female sex, obesity type II, and weight loss accounted for a reduced risk regarding the hazard of HF readmission, while the presence of obesity type I decreased the risks for both all‐cause and HF rehospitalizations. Detailed information on this is provided in *Table*s 3 and 4. The results of the sensitivity analysis in the PS‐matched subsample are similar to those from the multivariable Cox regression in the full cohort (all‐cause readmission: HR 0.77, 95% CI 0.72–0.81; HF readmission: HR 0.87, 95% CI 0.81–0.94).

**Table 3:** Effect of the four‐drug therapy and other variables on the hazard of rehospitalization for all‐cause 91–365 days after index stay

Risk factor	HR	95% CI
Four‐drug therapy (Ref. two‐drug therapy)	0.76	0.73–0.80
Patient age group (Ref. <66 years)		
66–70 years	1.05	0.997–1.11
71–75 years	1.05	1.00–1.09
76–80 years	1.14	1.09–1.19
81–85 years	1.22	1.17–1.27
86–90 years	1.29	1.24–1.35
>90 years	1.37	1.31–1.44
Comorbid conditions		
NYHA stage (Ref. NYHA IV)		
NYHA I	0.96	0.88–1.05
NYHA II	0.96	0.93–0.997
NYHA III	0.99	0.97–1.02
BMI (Ref. BMI < 30 kg/m^2^)		
BMI 30–34	0.94	0.89–0.99
BMI 35–39	1.02	0.96–1.08
BMI 40+	1.11	1.05–1.16
COPD	1.11	1.08–1.14
Diabetes, uncomplicated	1.07	1.04–1.09
Diabetes, complicated^a^	1.12	1.08–1.15
Renal failure	1.05	1.03–1.08
Year of hospital stay (Ref. 2019)		
2020	1.06	1.03–1.09
2021	1.03	1.01–1.06

Model was adjusted for year of hospital stay, patient age group, sex, NYHA stage (IV vs. I, II, III), BMI (<30 kg/m^2^ vs. 30–34, 35–39, and 40+ kg/m^2^), dementia, cardiogenic and unspecified shock, ischaemic stroke, intracerebral bleeding, and concomitant diseases at the index hospitalization by Elixhauser *et al*.^18^ Only significant risk factors are shown.

CI, confidence interval; COPD, chronic obstructive pulmonary disease; HR, hazard ratio; NYHA, New York Heart Association Functional Classification; Ref., reference.

^a^ Coma, ketoacidosis, vascular disease.

**Table 4:** Effect of the four‐drug therapy and other variables on the hazard of rehospitalization for HF 91–365 days after index stay

Risk factor	HR	95% CI
Four‐drug therapy (Ref. two‐drug therapy)	0.90	0.85–0.96
Patient age group (Ref. <66 years)		
66–70 years	1.13	1.04–1.22
71–75 years	1.19	1.10–1.28
76–80 years	1.35	1.26–1.44
81–85 years	1.51	1.42–1.61
86–90 years	1.66	1.55–1.78
>90 years	1.79	1.65–1.94
Female patient	0.92	0.89–0.95
Comorbid conditions		
NYHA stage (Ref. NYHA IV)		
NYHA I	0.59	0.50–0.69
NYHA II	0.71	0.67–0.76
NYHA III	0.96	0.93–0.99
BMI (Ref. BMI < 30 kg/m^2^)		
BMI 30–34	0.89	0.83–0.96
BMI 35–39	0.99	0.91–0.99
BMI 40+	1.17	1.09–1.26
Dementia	0.88	0.82–0.93
Peripheral vascular disorders	1.09	1.04–1.14
Pulmonary circulation disorder	1.13	1.08–1.18
Valvular disease	1.11	1.08–1.15
COPD	1.19	1.14–1.24
Cardiac arrhythmia	1.21	1.17–1.25
Diabetes, uncomplicated	1.16	1.12–1.20
Diabetes, complicated^a^	1.24	1.19–1.29
Renal failure	1.26	1.22–1.30
Weight loss	0.87	0.78–0.98

Model was adjusted for year of hospital stay, patient age group, sex, NYHA stage (IV vs. I, II, III), BMI (<30 kg/m^2^ vs. 30–34, 35–39, and 40+ kg/m^2^), dementia, cardiogenic and unspecified shock, ischaemic stroke, intracerebral bleeding, and concomitant diseases at the index hospitalization by Elixhauser *et al*.^18^ Only significant risk factors are shown.

CI, confidence interval; COPD, chronic obstructive pulmonary disease; HR, hazard ratio; NYHA, New York Heart Association Functional Classification; Ref., reference.

^a^ Coma, ketoacidosis, vascular disease.

## Discussion

This retrospective routine data study of 315 342 HF patients examined the prevalent prescription regimens in HF therapy and compared the associations of a quadruple‐drug treatment on mortality and readmission rates to that of the outdated two‐drug approach in the time span from 91 to 365 days after index hospitalization. Our results show significant benefits of the newly suggested ESC guideline therapy for both endpoints.

The median age of our study population was 80 years with slightly more females than males. This deviates from the age and sex distribution reported in earlier European HF studies. They, in turn, typically consisted of cohorts that were medially in their 60s and of a male sex in three‐quarters of the cases.^21^ A predominantly female representation is solely found in the sub‐group of HF with preserved ejection fraction (HFpEF).^22^ Evaluating the exact distribution of HF type is, however, not possible in our routinely collected patient data, which presumably consists of mainly HFrEF, because HFpEF cases are normally not hospitalized under the main diagnosis of HF. As the main hospital diagnoses are thoroughly externally controlled in Germany, we assume that HFpEF cases are masked among diabetics and hypertension admissions although this remains speculative. There is no specific ICD‐10 code to identify patients with HFpEF. Secondly, it must be considered that our cohort, without exception, includes patients that were initially in need of hospital admission and, therefore, were presumably sicker than those who have likewise been diagnosed with HF but remained outpatients. Accordingly, a comparison of our work with epidemiological data of solely hospitalized HF patients is much more precise. For instance, in a study by Fernández Gasso *et al*., age and sex distribution of a hospitalized cohort were almost congruent with our own results, seeing as sicker populations also tend to be older and, thus, predominantly female.^23^ The most common comorbidity in our study cohort was hypertension, with slightly more than 80% of patients being affected, followed by atrial fibrillation, kidney failure, and coronary heart disease. A similar profile of attributable co‐illnesses was detected in prior studies, however, with generally fewer comorbidities per patient^24^; the difference may simply be a result of the fact that our study cohort was of a higher age. Overall, we included 315 342 patients that had an index hospital stay for HF during our study period. Stratified by year, the number of patients declined by almost 16% between the first 2 years and only increased again slightly in 2021. It can be assumed that this decrease most probably resulted from the COVID‐19 pandemic, a period in which hospitalization and emergency department consultations for HF, but also other non‐COVID‐19‐related diseases, were reduced.^25^, ^26^, ^27^ For our follow‐up span, similar tendencies of a relatively low readmission rate were detectable. Various studies reported a comparatively higher number of HF rehospitalizations.^28^, ^29^ Nevertheless, rehospitalization for HF is the dominating cause in our study, and other issues are pretty divers (data not shown). As we deal with a routine population, other relevant diseases maybe more prevalent than in prospective studies due to exclusion criteria.

When looking at HF medication numbers in total and over time, we observed that the two oldest HF medication categories were among the most‐prescribed ones with BB reaching almost 81%, followed by ACEI and ARB. The combined two‐drug treatment was, in total, received by around 36% of all included patients, with a declining tendency over the years. While the newly endorsed HF medication combination was only yet prescribed in a minority of cases, at around 1–2% in the first two study years, a rise up to 7.7% was visible in 2021. When taking into account that the current ESC guideline was published in 2021,^2^ this slow but detectable increase seems plausible. Well‐founded evidence for the benefits of each individual new HF drug class was, however, already available prior to the renewal of official ESC guidelines. For instance, positive effects of MRAs on mortality and morbidity of cases with severe HF were reported as early as the late 1990s, starting with the RALES study.^5^ SGLT2is gained their attention regarding major benefits in the treatment of HF with the DAPA‐HF and EMPEROR‐Reduced trials, which were published in 2019 and 2020.^9^, ^30^ This explains the presence, albeit minimally, of quadruple therapy prescriptions in the first 2 years of observation prior to the publication of official recommendations. When taking a precise look at the sub‐group that did receive GDMT, it is noticeable that they primarily consisted of males, and of patients aged <66 years. This could be the result of the physicians' tendency to prescribe the newly endorsed treatment especially to a young population at initial diagnosis and not to older HF patients whose symptoms may already seem adequately controlled with the existing pharmacological treatment strategy. The lower utilization in women could be gender bias. In general, 72.5% of our study population received either a two‐, three‐, or four‐drug therapy regimen for HF. Reasons for non‐utilization of GDMT or the use of alternative combination treatments can only be speculated upon and not be evaluated based on routinely collected pharmacological data. Prior research analysing this issue has shown missing ACEI/ARB and MRA prescriptions in one‐ to two‐thirds, respectively. Problems in initiation and titration are considered as major reasons for non‐utilization,^31^ showing that therapeutic guideline adherence does not solely depend on patient factors but also on the physician's decision to follow prescription suggestions despite personal insecurities. Regarding additional HF therapy, it was remarkable that over 90% of the HF cases also had a diuretic co‐prescribed. In other HF studies, rates were also high, though mostly around 80%.^15^ A possible add‐on benefit of diuretic use on the endpoints' morbidity, mortality, and readmission has been discussed by Faris *et al*.; however, it is yet only scarcely investigated.^32^

Death was reported in almost 18% of our HF population and, therefore, roughly in line with existing data sets of other studies.^33^ For patients with HF, it is known that the risk of mortality rises with a hospital stay.^34^ The factors that increased the mortality risk in our population reflect the results of existing literature and included, in particular, male sex, higher age, and (cardiovascular) comorbidities.^35^, ^36^ Obesity stages I and II were, on the contrary, associated with a lower risk of death. This observation is in accordance with research that has reported a so‐called obesity paradox in HF patients, where moderate obesity accounts for higher survival rates than seen in normal or underweight.^37^ As hypothesized, the survival rate was significantly higher during the observation span for patients who had received the GDMT than for those with a prescription for the two‐drug standard. This corresponds to the findings of the cross‐trial analysis published by Vadugnathan *et al*., who likewise compared these two combination regimens, however, based on data from existing single‐drug RCTs.^14^ Our sub‐group analysis further showed that the beneficial effect on mortality minimized with increasing age and that it was not significant in patients between 66 and 85 years old, while positive effects on readmission rates remained stable for all age categories. These results are partly in line with findings from Vaduganathan *et al*., who described a similar age‐dependent difference in the combined endpoint of cardiovascular death, however, also for readmission.^14^ For both endpoints, no sex‐dependent difference in outcome between two‐ and four‐drug treatment was observed. Interestingly, survival rates were also significantly higher in patients that received a three‐drug therapy versus patients with a two‐drug prescription. In cases where patients may have contraindications or experience significant side effects in the use of either MRAs or SGLT2is, a visible improvement of outcome can, therefore, also be expected if only three of the four major HF drug classes are combined. Komajda *et al*. performed a network meta‐analysis that indicated similarly significant benefits for the triple combined therapy of ARNI, BB, and MRA and, thus, support our results.^13^

Readmission analysis in our work indicated a significantly better outcome for people with a quadruple therapy than compared to the old standard treatment protocol, with regard to all‐cause and HF rehospitalization. This is in line with results of prior RCTs on the basis of which ESC guidelines were redeveloped in 2021.^2^ Factors attributed to a significantly higher risk for the hazard of rehospitalization were similar to those that were associated with a higher mortality risk.

### Strengths and limitations

Our routine data study is the first of its kind that retrospectively analyses a large HF cohort of 315,342 patients regarding the associations and possible benefits of the four‐drug therapy standard. With this, it adds highly interesting real‐world information to the already existing RCTs. Nevertheless, we assume that our data set mainly consists of patients with reduced EF, but information on EF is unknown, and results of exact HF categories are not available. In addition, the study was conducted during the COVID‐19 pandemic that could have influenced the outcome since patient follow‐up visits were restricted, leading potentially to worse compliance and lower drug adherence and persistence. Although observational routine data cannot account for causal inference, the large case numbers allow to see signals in routine use, which are positive and in line with actual RCT results. While patient numbers in our study may be sufficient to draw first conclusions, a larger and updated future data set is needed in which increasing prescriptions of the GDMT can be assumed. Although our study is based on nationwide data of the largest provider of healthcare insurance in Germany, there may be variations in terms of age, sex, social status, and morbidity between patients insured by different German healthcare providers.^38^ For instance, comparing AOK cases to all German patients with HF in 2021, there are slight differences to our study population (female sex 53.5% [AOK] vs. 50.0% [Germany], age ≥80 years 60.1% [AOK] vs. 59.5% [Germany]).^39^ Furthermore, there were substantial differences in the patient populations between the two‐drug and the relatively small four‐drug therapy groups. Even though our analysis was risk‐adjusted for age, gender, and comorbidities and a propensity‐matched sensitivity analysis supported the hypothesis that the new four‐drug standard therapy is beneficial, there may be additional unmeasured confounders. Also, it must be noted that the use and dosage of medications is not reported in detail, which is a limitation of our database. As commonly the case for retrospectively collected pharmacological data, there is a level of uncertainty between the prescription of a drug and actual adherence. Side effects, for instance, can lead to a reduction in dose or a complete discontinuation of intake. Therefore, effects of prescription data on outcome should be interpreted cautiously.

Finally, our data show the potential benefits of the newly recommended four‐drug therapy standard, but not the impact of the actual guideline on the implementation of this standard. Future studies will be done to address, whether the guideline led to an increase of patients treated with this new standard and if this increases the benefits.

### Conclusions

This present report suggests a superiority of the GDMT concerning survival rate and all‐cause/HF readmission in the first year after index hospitalization for HF. With this, our work clearly supports current ESC guideline practice and findings of prior RCTs. Our findings need to be verified in updated and larger real‐world analysis. At the same time, reasons for non‐utilization should be identified to improve therapy adherence.

## Conflict of interest

Outside of this study, M.M. received speakers and consulting fees from Bayer Healthcare, BMS, Boehringer Ingelheim, Daiichi Sankyo, Astra Zeneca, Sanofi, BRAHMS GmbH, and Roche Diagnostics as well as research funding from German public funding authorities for Health Care Research and Roche Diagnostics; S.P. received research funding from Roche Diagnostics; G.M. received speaker fees from Getinge, Orion Pharma, and AOP Orphan Pharmaceuticals Germany GmbH.

## Funding

This work was not supported by any funding.

## Supporting information


**Figure S1.** Venn‐Diagrams of drug use in HF patients.
**Table S1.** Prescription of drug combinations per year.
**Table S2.** Comparison of baseline characteristics between subjects treated with four‐drug combination and two‐drug combination in the full cohort and in the propensity score matched sample.

Supporting info item
