# Supplementary material for: Prescription of guideline‐directed medical therapy in heart failure: impact on mortality and readmission
Source: ESC Heart Fail. 2025 Apr 29;12(4):2791–802. doi: 10.1002/ehf2.15280 (PMC12287797; doi:10.1002/ehf2.15280)
Supplement: Supplementary file 1 — Figure S1. Venn‐Diagrams of drug use in HF patients. Table S1. Prescription of drug combinations per year. Table S2. Comparison of baseline characteristics between subjects treated with four‐drug combination and two‐drug combination in the full cohort and in the propensity score matched sample. [file EHF2-12-2791-s002.docx]

**Appendices**

**Supplementary Figure 1. Venn-Diagrams of drug use in HF patients.**

Venn-Diagram showing the distribution of HF medication use and the overlap of combined drug therapies. The blue square accounts for all 315,342 included HF patients. 70,450 (22.24%) of the population does not receive the combination ACEI-ARB/ARNI + BB and/or MRA and/or SGLT2i. 12,874 (4.08%) did not have a prescription of any single drug of the above mentioned.

Abbreviations: ACEI Angiotensin-converting enzyme inhibitor; ARB Angiotensin receptor blocker; ARNI Angiotensin receptor-neprilysin inhibitor; BB beta-blocker; MRA mineralocorticoid receptor antagonist; SGLT2i sodium-glucose co-transporter 2 inhibitor.

**Supplementary Table 1. Prescription of drug combinations per year.**

| **Year** | **2019-2021** | | **2019** | | **2020** | | **2021** | |
| --- | --- | --- | --- | --- | --- | --- | --- | --- |
| **Total** | **N** | **%** | N | % | N | % | N | % |
|  | **315,342** | **100.00%** | 116,91 | 100.00% | 98,650 | 100.00% | 99.785 | 100.00% |
| **Two-drug combination ACEI/ARB + BB** | **113,341** | **35.94%** | 44,232 | 37.84% | 35,94 | 36.43% | 33,17 | 33.24% |
| **Four-drug combination: ACEI/ARB/ARNI + BB + MRA + SGLT2i** | **11,553** | **3.66%** | 1,492 | 1.28% | 2,344 | 2.38% | 7,717 | 7.73% |
|  |  |  |  |  |  |  |  |  |
|  |  |  |  |  |  |  |  |  |
| **Three-drug combination: ACEI/ARB/ARNI + BB + MRA** | **84,815** | **26.90%** | 30,123 | 25.77% | 26,56 | 26.92% | 28,13 | 28.19% |
| **Three-drug combination: ACEI/ARB/ARNI + BB + SGLT2i** | **18,884** | **5.99%** | 2,841 | 2.43% | 3,967 | 4.02% | 12,08 | 12.10% |

Distribution of HF drug prescription in the study observation span. Stratification was performed based on the prescription of a two-drug (ACEI/ARB + BB), three-drug (ACEI/ARB/ARNI + BB + MRA or ACEI/ARB/ARNI + BB + SGLT2i) or four-drug (ACEI/ARB/ARNI + BB + MRA + SGLT2i) combination.

Abbreviations: ACEI Angiotensin-converting enzyme inhibitor; ARB Angiotensin receptor blocker; ARNI Angiotensin receptor-neprilysin inhibitor; BB beta-blocker; MRA mineralocorticoid receptor antagonist; SGLT2i sodium-glucose co-transporter 2 inhibitor.

**Supplementary Table 2. Comparison of baseline characteristics between subjects treated with four-drug combination and two-drug combination in the full cohort and in the propensity score matched sample.**

| Endpoint Death (91-365d) | Full cohort | | | Matched sample (caliper matching) | | |
| --- | --- | --- | --- | --- | --- | --- |
| Patient characteristics | Four-drug therapy  (n=11.553) | Two-drug therapy  (n=113.341) | Standardized difference | Four-drug therapy  (n=11.553) | Two-drug therapy  (n=11.553) | Standardized difference |
| Age (y) | 69.9 ± 11.8 | 79.6 ± 9.9 | -0.883 | 69.9 ± 11.8 | 70.0 ± 12.8 | -0.008 |
| Female sex | 30.8% | 60.7% | -0.628 | 30.8% | 29.4% | 0.043 |
| BMI (kg/m²) |  |  |  |  |  |  |
| 30-34 | 5.8% | 5.0% | 0.036 | 5.8% | 5.1% | 0.029 |
| 35-39 | 4.3% | 3.4% | 0.046 | 4.3% | 4.3% | 0.016 |
| ≥ 40 | 6.6% | 4.2% | 0.106 | 6.6% | 6.4% | 0.020 |
| Left heart failure |  |  |  |  |  |  |
| NYHA I | 0.9% | 2.0% | -0.930 | 0.9% | 0.7% | 0.015 |
| NYHA II | 6.6% | 10.2% | -0.130 | 6.6% | 6.1% | 0.013 |
| NYHA III | 46.3% | 41.6% | 0.093 | 46.3% | 46.6% | -0.011 |
| NYHA IV | 45.4% | 35.9% | 0.193 | 45.4% | 45.7% | 0.004 |
| Hypertension | 77.6% | 86.0% | -0.221 | 77.5% | 77.5% | 0.012 |
| Atrial fibrillation/flutter | 47.5% | 56.1% | -0.172 | 47.5% | 49.0% | -0.033 |
| Coronary heart disease | 64.2% | 40.8% | 0.483 | 64.2% | 64.4% | -0.011 |
| Acute myocardial infarction | 4.9% | 2.6% | 0.121 | 4.9% | 4.7% | -0.006 |
| Prior myocardial infarction | 13.1% | 7.4% | 0.189 | 13.1% | 13.3% | 0.019 |
| Mitral valve disorder | 23.0% | 14.4% | 0.223 | 23.0% | 22.4% | 0.017 |
| Aortic valve disorder | 9.0% | 11.3% | -0.077 | 9.0% | 8.9% | -0.017 |
| Dilated cardiomyopathy | 24.0% | 3.9% | 0.606 | 24.0% | 24.2% | 0.002 |
| Prior stroke or intracranial bleeding | 1.6% | 2.8% | -0.080 | 1.6% | 1.5% | 0.003 |
| Ventricular tachycardia | 3.8% | 0.7% | 0.207 | 3.8% | 3.6% | 0.009 |
| Renal failure | 35.6% | 50.2% | -0.298 | 35.6% | 37.8% | -0.026 |
| Diabetes | 66.3% | 39.7% | 0.554 | 66.3% | 65.8% | 0.012 |
| COPD | 16.6% | 15.7% | 0.026 | 16.6% | 16.6% | 0.003 |
| Pneumonie | 11.0% | 12.0% | -0.031 | 11.0% | 10.6% | 0.032 |
| Depression | 4.1% | 5.8% | -.0764 | 4.1% | 3.6% | 0.026 |
| Dementia | 2.4% | 8.0% | -0.257 | 2.4% | 2.1% | 0.009 |
| Solid tumor without metastasis | 1.4% | 1.9% | -0.039 | 1.4% | 1.3% | 0.006 |
| Lymphoma | 0.2% | 0.3% | -0.008 | 0.2% | 0.2% | 0.000 |
| Metastatic cancer | 0.4% | 0.5% | -0.011 | 0.4% | 0.3% | -0.007 |
| Paralysis | 1.7% | 2.3% | -0.046 | 1.7% | 1.4% | 0.005 |
| Rheuma | 1.3% | 2.1% | -0.066 | 1.3% | 1.3% | 0.009 |
| Coagulopathy | 2.8% | 2.5% | 0.018 | 2.8% | 2.3% | 0.009 |
| Weight loss | 1.6% | 2.1% | -0.033 | 1.6% | 1.5% | 0.021 |
| Fluid and electrolyte disorders | 30.4% | 32.3% | -0.042 | 30.4% | 30.7% | 0.002 |
| Iron deficiency anemia | 6.7% | 7.2% | -0.019 | 6.7% | 6.5% | 0.009 |
| Neurological Disorders | 2.1% | 4.1% | -0.110 | 2.1% | 1.8% | -0.001 |
| Peripheral vascular disorders | 14.9% | 12.0% | 0.085 | 14.9% | 14.4% | 0.028 |
| Pulmonary circulation disorder | 16.1% | 15.5% | 0.016 | 16.1% | 14.9% | 0.050 |
| Alcohol abuse | 2.5% | 0.9% | 0.117 | 2.5% | 2.4% | 0.012 |
| Psychosis | 0.7% | 0.5% | 0.029 | 0.7% | 0.5% | 0.016 |

Continuous variables are reported as means ± standard deviations. Dichotomous variables are reported in percent. The propensity score matching sample was constructed by nearest neighbour matching (1:1) on the logit of the propensity score using calipers of width equal to 0.2 of standard deviation of the logit of the propensity score. The standardized differences were greatly reduced after matching.

Abbreviations: COPD chronic obstructive pulmonary disease; NYHA New York Heart Association Functional Classification
