# Supplementary figures and images for: Prescription of guideline‐directed medical therapy in heart failure: impact on mortality and readmission
Source: ESC Heart Fail. 2025 Apr 29;12(4):2791–802. doi: 10.1002/ehf2.15280 (PMC12287797; doi:10.1002/ehf2.15280)

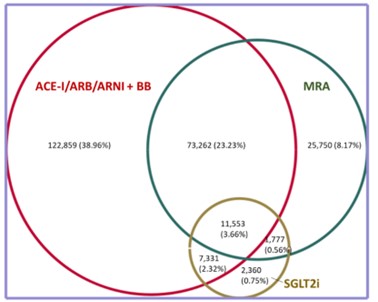

Supplement: Supplementary file 2 — Supporting info item [file EHF2-12-2791-s001.jpg]
